# Cadherin-23 May Be Dynamic in Hair Bundles of the Model Sea Anemone *Nematostella vectensis*

**DOI:** 10.1371/journal.pone.0086084

**Published:** 2014-01-22

**Authors:** Pei-Ciao Tang, Glen M. Watson

**Affiliations:** Department of Biology, University of Louisiana at Lafayette, Lafayette, Louisiana, United States of America; Claremont Colleges, United States of America

## Abstract

Cadherin 23 (CDH23), a component of tip links in hair cells of vertebrate animals, is essential to mechanotransduction by hair cells in the inner ear. A homolog of CDH23 occurs in hair bundles of sea anemones. Anemone hair bundles are located on the tentacles where they detect the swimming movements of nearby prey. The anemone CDH23 is predicted to be a large polypeptide featuring a short exoplasmic C-terminal domain that is unique to sea anemones. Experimentally masking this domain with antibodies or mimicking this domain with free peptide rapidly disrupts mechanotransduction and morphology of anemone hair bundles. The loss of normal morphology is accompanied, or followed by a decrease in F-actin in stereocilia of the hair bundles. These effects were observed at very low concentrations of the reagents, 0.1–10 nM, and within minutes of exposure. The results presented herein suggest that: (1) the interaction between CDH23 and molecular partners on stereocilia of hair bundles is dynamic and; (2) the interaction is crucial for normal mechanotransduction and morphology of hair bundles.

## Introduction

Hair bundles are the mechanoreceptor apparatus of hair cells. Hair bundles consist of stereocilia, evaginations of the plasma membrane filled with actin filaments. Stereocilia are graded in length across the hair bundle to form a staircase array [1]. Invariably, hair bundles transduce signals when the hair bundle is deflected. Mechanotransduction channels in stereocilia of hair bundles are responsible for converting mechanical stimulation into electrical/chemical signals that ultimately result in such phenomena as hearing and balance [2]–[4]. Mechanotransduction channels appear to be located distally in stereocilia. An extracellular current was measured near the distal end of moving hair bundles [5]. Later experiments performed with the iontophoretic application of gentamicin, an aminoglycoside antibiotic that blocks mechanotransduction, also indicated that the mechanotransduction channels are likely to be located near the tips of the stereocilia [6]. Finally, mechanotransduction channels were directly localized to the tips of stereocilia with calcium imaging in hair cells from bullfrogs [7] and from rats [8]. Based on the relationship between hair bundle displacement and the mechanotransduction current, it was proposed that an elastic element is attached to the mechanotransduction channel [9], [10]. According to the gating spring model, when a hair bundle is deflected in a positive direction (i.e., toward the taller stereocilia), mechanotransduction channels open rapidly to allow a cation influx. Because stretching the gating-spring raises the energy level of the closed state, the mechanotransduction channel's open state is energetically favored. At rest, an estimated 15–20 percent of the mechanotransduction channels are open because of significant resting tension on the gating spring. Therefore, the resting mechanotransduction current was observed at 20∼40% of maximum instead of at zero [11].

Stereocilia are interconnected by several types of extracellular linkages, including ankle links, lateral links, and tip links which collectively cause stereocilia to move as a unit. Of particular interest are tip links that interconnect adjacent stereocilia by extending from the tips of shorter stereocilia to the sides of the adjacent taller stereocilia [12]. Initially, tip links were proposed to serve as the gating spring [12], [13]. However, analyses of electron micrographs suggested that tip links consist of helically wound filaments, forming a structure that is unlikely to be elastic, as predicted for the gating spring [14], [15]. Thus, recent models suggest that tip links lie in series with the gating spring and the mechanotransduction channel [4].

Two known protein components of tip links are cadherin 23 (CDH23) and protocadherin 15 (PCDH15) [16], [17]. CDH23 was localized to tip links in a variety of vertebrate animals, including zebrafish [18], bullfrogs [16], and guinea pigs [17]. The CDH23 polypeptide encompasses three parts; an ectodomain, a transmembrane domain, and a C-terminal cytoplasmic domain. The predicted organization of the CDH23 ectodomain is similar to that for classic cadherins [19]. However, an extended ectodomain in CDH23 is not found in the classic cadherins. Hair cells undergo a rapid loss of tip links and lose mechanosensitivity after they are exposed to calcium-depleted buffers containing such chelators as EGTA or BAPTA [20], [21]. Evidently, calcium ions are required to maintain CDH23 structure and rigidity [22].

Interestingly, vertebrate-style hair bundles occur in sea anemones, marine invertebrates belonging to the phylum, *Cnidaria* [23]. In sea anemones, hair bundle mechanoreceptors on the tentacles detect swimming movements of nearby prey [24]. Stimulated hair bundles excite the anemone such that objects that touch the tentacles are maximally stung by nematocysts. In the absence of vibrations, or in the presence of vibrations at frequencies other than key frequencies, approximately half as many nematocysts are discharged [24], [25]. Anemone hair bundles are unusual insofar as their morphology and responsiveness are adjustable according to the activity of specific chemoreceptors that bind prey-derived compounds. For example, activated chemoreceptors for N-acetylated sugars induce the hair bundles to elongate [26] while shifting maximal discharge of nematocysts downward to key frequencies overlapping those produced by calmly swimming prey [24], [25]. On the other hand, anemone hair bundles are conventional in that mechanotransduction is reversibly blocked by aminoglycoside antibiotics and lost following brief exposure to calcium-depleted seawater [27]. Moreover, anemone hair bundles consist of actin-based stereocilia interconnected by linkages including tip links [23]. A CDH23-like polypeptide was localized by immunocytochemistry to stereocilia of hair bundles of sea anemones [28]. Phylogenetic analysis indicated that the CDH23-like polypeptide in sea anemones is a homolog of CDH23 in vertebrates [28]. However, the predicted anemone polypeptide is large, including three membrane-spanning alpha helices and 44 CDH repeat domains [28]. As a consequence, the N-terminus is predicted to lie in the cytoplasm while a small C-terminal domain is predicted to lie on the exoplasmic side of the plasma membrane. In this study, we investigated the effects of masking the unusual C-terminus of CDH23 or of mimicking the C-terminus on hair bundle structure and function in the model sea anemone *Nematostella vectensis*.

## Materials and Methods

### Ethics statement

Scientific research on sea anemones does not require approval by the IACUC. Nevertheless, anemones were fully anesthetized in tricaine/potassium-enriched seawater prior to dissection or fixation.

### CDH23 peptide and CDH23 antibody

A CDH23 peptide ((C)SEMDMTYDAYRYDETTL) corresponding to residues 6058–6074 in a C-terminal motif in a *N. vectensis* cadherin was synthesized and then used as an antigen in the production of polyclonal antibodies in chickens [28]. The CDH23 peptide sequence is unique to *N. vectensis* based on the results of blast to the NCBI database (http://www.ncbi.nlm.nih.gov/) and the StellaBase database (http://nematostella.bu.edu/stellabase/). Immunocytochemistry employing the affinity-purified CDH23 antibody labeled stereocilia of hair bundles of sea anemones. Furthermore, immunoelectron microscopy labeled stereocilia in the vicinity of insertion points of tip links. Immunolabeling was abolished when the primary antibody was pre-incubated with the peptide antigen [28]. For this study, the peptide and its corresponding, affinity-purified antibody were applied to seawater containing intact anemones or isolated tentacles in order to test their effects on hair bundle structure and function. In addition, the antibody was employed in immunocytochemistry performed at the light microscopic level to confirm normal biological activity of the frozen, stored antibodies. Finally, the cadherin-23-like peptide was conjugated with fluorescein isothiocyanate (FITC) (Biomatik USA LLC., DE, USA) in order to localize its binding partners in sea anemone hair cells.

### *N. vectensis* CDH23 and TRPN1 inmmunocytochemistry

Five specimens of *N. vectensis* were anesthetized in tricaine/potassium-enriched seawater (2% tricaine, 323 mM NaCl, 26 mM MgSO_4_, 24 mM MgCl_2_, 100 mM KCl, 12 mM CaCl_2_, and 2 mM NaHCO_3_) for 15 min. The oral discs including the array of tentacles were excised with a scalpel and fixed in 0.025% glutaraldehyde and 4% paraformaldehyde in Sorensen's phosphate buffer (199 mM Na_2_HPO_4_, 48 mM NaH_2_PO_4_, and 150 mM NaCl) for 45 min at RT. The fixed tissue was washed in 0.1 M glycine in PBS for 5 min followed by three times in PBS, 5 min each. The tissue was incubated in 3% BSA/PBS for 30 min at RT in order to block non-specific binding of the primary antibody. Subsequently, the tissue was incubated in 1/100 dilution of the affinity-purified CDH23 antibody at 4°C overnight. On the next day, the tissue was washed in PBS three times (5 min each) before incubation in a 1/500 dilution of secondary antibody (goat anti-chicken IgG conjugated to Alexa Fluor® 555) at RT for 1 hour in dark. After the incubation of the secondary antibody, the specimens were washed three times in PBS (5 min each). Tentacles were excised with a scalpel, prepared as wet mounts in which Vaseline posts were incorporated at the corners of the coverslip to prevent compression of the tissue, and imaged under epifluorescence microscopy (model RP011-T, LOMO America, IL, USA) with a 100X oil immersion objective (na = 1.30, LOMO America, Inc., IL, USA). Images were captured with a STL-11000M SBIG cooled CCD camera (SBIG, CA, USA) operated using Maxim-DL software (Diffraction Limited, ON, Canada). Brightfield microscopy was performed using oblique contrast as described [28] Overlays of images obtained using oblique contrast and epifluorescence microscopy were generated using Maxim-DL software. In addition, immunocytochemistry was performed to localize a homolog of TRPN1 in the sensory epithelium of *Nematostella*. The homolog was identified in the StellaBase (SB56566). The specific peptide antigen ((C)RKQPDRLHTGYQPRQRR) corresponds to residues 1170–1186 near to the C-terminus of the TRPN1 polypeptide. The peptide sequence is unique to the TRPN1 homolog in *Nematostella*. Affinity purified antibodies raised to this peptide were employed in immunocytochemistry using the methods detailed above.

### *N. vectensis* CDH23 peptide cytochemistry

Five specimens of *N. vectensis* were anesthetized in tricaine/potassium-enriched seawater for 15 min. Then, the dissected oral discs including the tentacle array were incubated in tricaine/potassium-enriched seawater containing 200 µg/ml CDH23 peptide-FITC for 30 seconds at room temperature (RT) in the dark followed by a rinse in potassium-enriched seawater. The tissue was fixed in 0.05% glutaraldehyde and 4% paraformaldehyde in Sorensen's phosphate buffer for 45 min at RT in the dark. After fixation, the tissue was washed 3times in PBS, each for 5 min. Tentacles were excised with a scalpel, prepared as wet mounts in which Vaseline posts were incorporated at the corners of the coverslip to prevent compression of the tissue, and imaged under epifluorescence microscopy as described above.

### *N. vectensis* vibration sensitivity bioassay

Vibration sensitivity bioassays were conducted on intact anemones followed the procedure described in [29] with minor modifications. Briefly, sea anemones were transferred from mass culture dishes to petri dishes filled with 30 ml of seawater at ½ dilution (∼16 ppt) and allowed 30 min to recover from handling. Vibrations were produced by a piezo bimorph (model EPA-007-612; Piezo System Inc., MA, USA) driven by a digital function generator (modelSG-100; Telulex Inc., CA, USA). A glass capillary attached to the piezo strip was lowered into the dish approximately 1 cm away from the anemone tentacles. After subjecting the anemones to vibrations at the key frequency of 56 Hz for several seconds, a small piece of fishing line coated with 25% gelatin (hereafter referred to as the “test probe”) was used to touch tentacles. The gelatin coat was rehydrated in the seawater for 5 sec before the touch such that nematocysts become embedded in the gelatin coating as they discharge. Each test probe was touched to a separate anemone. Test probes were pulled away from the anemone and then fixed in 2.5% glutaraldehyde in seawater for a minimum of 30 min. Fixed probes were prepared as wet mounts and examined using phase contrast microscopy. Patches of discharged nematocysts on the test probe correspond to areas of the probes touched by anemone tentacles. A single representative field of view at 400X magnification was selected for a single patch of nematocysts for each probe imaged. Basitrichous isorhiza (i.e., basitrich) nematocysts were counted. Approximately twice as many nematocysts are discharged into test probes touched to tentacles at key frequencies as compared to other frequencies, or in the absence of vibrations. Accordingly, levels of discharge obtained in the absence of nearby vibrations are designated as ‘baseline’discharge. Likewise, (in the presence of vibrations at a key frequency), levels of discharge higher than baseline are referred to as ‘vibration-dependent discharge.’

Following the 30 min recovery period, experimental anemones were incubated in seawater fortified with 0.1 nM affinity-purified CDH23 antibody. The CDH23 antibody solution was added by pipetting from a stock solution into the dish. Every treatment was tested with 10 to 15 replicate test probes. Vibration sensitivity was assayed at 5, 10, 30, and 60 min after the CDH23 antibody was added. In order to exclude the possibility that any decrease in nematocyst discharge results from the presence of an antibody in general, an affinity purified TRPA1 antibody was used as an antibody-loading control. The TRPA1 antibody was prepared in chickens to a synthetic peptide ((C)DAFKRSTDNKL) corresponding to residues 750–761 in the cytoplasmic C-terminus of a homolog of TRPA1 in *N. vectensis* [30]. The peptide sequence was unique to the TRPA1 sequence (SB17686) in the online *N. vectensis* genome database, StellaBase. The epitope that binds the TRPA1 antibody is predicted to reside in the cytoplasm. Therefore, no effect of this antibody on vibration-dependent discharge of nematocysts was expected.

Vibration sensitivity bioassays also were performed on anemones before and after incubating anemones in 0.1 or 10 nM solutions of the CDH23 peptide at RT. Six replicate experiments were performed for each treatment. Vibration sensitivity was tested at specific time points after the peptide was added to the dish containing the anemones. In order to test the possibility that any decrease in vibration-dependent discharge of nematocysts might result from the addition of a peptide in general, a peptide-loading control was used in which a peptide ((C)YSKKAEEKEKQRREKAKEE) corresponding to residues 320–336 of a homolog in *N. vectensis* of harmonin (XP_001639780) was added to the seawater at a concentration of 10 nM. Harmonin is anticipated to reside in the cytoplasm. Hence, no effect of this peptide on vibration-dependent discharge of nematocysts was expected. The vibration sensitivity data were analyzed with repeated measures ANOVA to determine the effect of treatment (vibration control, non-vibration control, and antibodies or peptide treatment) and time on nematocyst discharge. Fisher's post-hoc tests were used to test for significant differences between certain treatments (*p*<0.05; STATISTICA software, StatSoft, Inc., OK, USA).

### Hair bundle morphology and abundance

Anemone hair bundles are conical in shape [31]. Hair bundles are composed of one true kinocilium located in the center and surrounded by a single circlet of large diameter stereocilia followed by several outer circular arrays of small diameter stereocilia [23], [29]. Whereas the kinocilium and large diameter stereocilia originate from a sensory neuron, the small diameter stereocilia originate from hair cells [23], [29]. To explore the possibility that exogenously added antibodies to CDH23, or added CDH23 peptides disrupt normal morphology, hair bundles were imaged with and without the addition of the antibodies (or peptides). The diameter of the tips and bases were measured from micrographs and the tip/base ratio of hair bundles was used to estimate the shape of hair bundles. After treatment with 0.1 nM CDH23 antibody or CDH23 peptide (0.1 and 10 nM), the oral discs of sea anemones were removed and fixed in 4% paraformaldehyde/0.05% glutaraldehyde in Sorensen's phosphate buffer for 45 min. After fixation, tissue was washed three times in PBS for 5 min each. The tentacles were then removed, prepared as wet mounts, and observed by oblique microscopy as described above. The diameters of the tips and bases of the hair bundles were measured from photomicrographs using ImageJ software (National Institutes of Health, MD, USA). The ratio of tip to base of each hair bundle was then calculated. Typically, five to eight anemones were imaged for each treatment.

In addition, the abundance of hair bundles was determined using phase contrast microscopy at 400X magnification. Three to four tentacles were assessed from each of the replicates from each treatment. The data were analyzed with repeated measures ANOVA to determine the effect of treatment and time on the abundance of hair bundles. Fisher's post-hoc tests were employed to test for significant differences between certain treatments (*p*<0.05; STATISTICA software, StatSoft, Inc., OK, USA).

### Electrophysiology

The methods employed for electrophysiological experiments were modified from those described in [27]. Briefly, anemones were anesthetized in artificial tricaine/potassium-enriched seawater for 15 min. After the anemones were fully anesthetized, one tentacle was removed and threaded using a fine human hair. Threaded tentacles then were transferred to a recording dish containing 5 ml of potassium-enriched seawater and the free ends of the hair were glued to the dish using a silicone elastomer (Kwik-Sil Adhesive, World Precision Instruments, FL, USA).

The experiments were performed on an inverted microscope (Model IMT-2, Olympus, Japan) mounted to an air table and enclosed by a Faraday cage (Model 63-531, Technical Manufacturing Corp., MA, USA). The recording dish was placed at a position that allowed hair bundles to be accessed by both the puffer and recording pipets. The puffer pipet (P_p_), was filled with 0.22 µm-filtered potassium-enriched seawater, and connected to a picopump pressure injector (model PV830, World Precision Instruments, FL, USA). The hair bundle was deflected by pressure jets delivered by the P_p_.

Recording pipets (P_r_) were pulled from 1.5 mm thick-walled glass pipets to form 0.2–0.3 µm diameter tips. The P_r_ was filled with 0.22 µm-filtered potassium-enriched seawater and coated with dimethylpolysiloxane to generate a high resistance seal. A patch clamp amplifier (Axopatch 200A, CV201AU headstage, Axon Instruments, CA, USA) was used in voltage-clamp mode with the pipet potential set to 0 mV, the gain set to 50 mV/pA, and an analog filter set to 1 kHz. During the recording, samples were monitored using video imaging (AVA StellaCam EX, NY, USA).

Recordings of membrane current were collected before and after perfusion with potassium-enriched seawater in order to test whether perfusion alone might disrupt mechanotransduction (perfusion control). Membrane current was recorded from hair bundles subjected to deflection. Ten cells were recorded as replicates. Each recording included two strong stimuli of 45 ms duration that deflected the tip of the hair bundle through 90 degrees. The puffer pipet was positioned at about one hair bundle distance (approximately 7 microns) from the hair bundle that was stimulated/recorded. To test the effect of CDH23 peptide on the response of the anemone hair bundles, 10 ml of potassium-enriched seawater with 10 nM CDH23 peptide (final concentration) was perfused into the recording dish while suction was applied to remove the pre-existing seawater from the dish. The perfusion required approximately 2 min to be completed. Mechanotransduction was tested before perfusion and then at 3 and 5 min after perfusion. Maximum current (peak current) induced by deflection was measured from ten hair bundles for each treatment including controls. The data were analyzed by repeated measures ANOVA to determine the effects of perfusion and/or the peptide addition over time. Fisher's post-hoc tests were used to compare means among specific treatments with significant differences reported at *p*<0.05.

### F-actin cytochemistry

Rhodamine phalloidin was used to label F-actin in stereocilia of hair bundles. Anemones were anesthetized in tricaine/potassium-enriched seawater. Healthy control and 10 nM CDH23 peptide treated samples were collected at 3, 5, and 30 min after the addition of CDH23 peptide. Three anemones were used for each treatment. The oral discs of anemones including the tentacle arrays were removed and fixed for 45 min in 4% paraformaldehyde/0.05% glutaraldehyde in the Sorensen's phosphate buffer. Fixed tissue was washed three times for five min each in PBS. The oral discs were then incubated in rhodamine phalloidin (Molecular Probes, OR, USA) at 4°C overnight. On the next morning, tissue samples were washed 3× each in PBS for 5 min. Then tentacles were excised and prepared as wet mounts with Vaseline posts at the corners of the coverslips to prevent compression of the tentacles. The tentacles were imaged as was described above. Exposure time was kept consistent. The fluorescence intensity of rhodamine phalloidin labeling was measured in grey values from 16 bit digital images using ImageJ software (National Institutes of Health, MD, USA). An area of interest was positioned over stereocilia that were favorably oriented in the image (lying flat). Mean fluorescence intensity was determined for the area of interest of the hair bundle. At this point, background fluorescence was measured by obtaining mean fluorescence intensity from a small area traced around the perimeter of the hair bundle. For each hair bundle, net fluorescence intensity was obtained by subtracting background fluorescence from the original value. A total of ten to twelve hair bundles was measured from two tentacles for each of the three anemones for each treatment (n = 3). Data were statistically analyzed using a one-way ANOVA followed by Fisher's Post hoc test.

## Results

### CDH23 antibodies bind to anemone hair bundles

**Figure 1:**
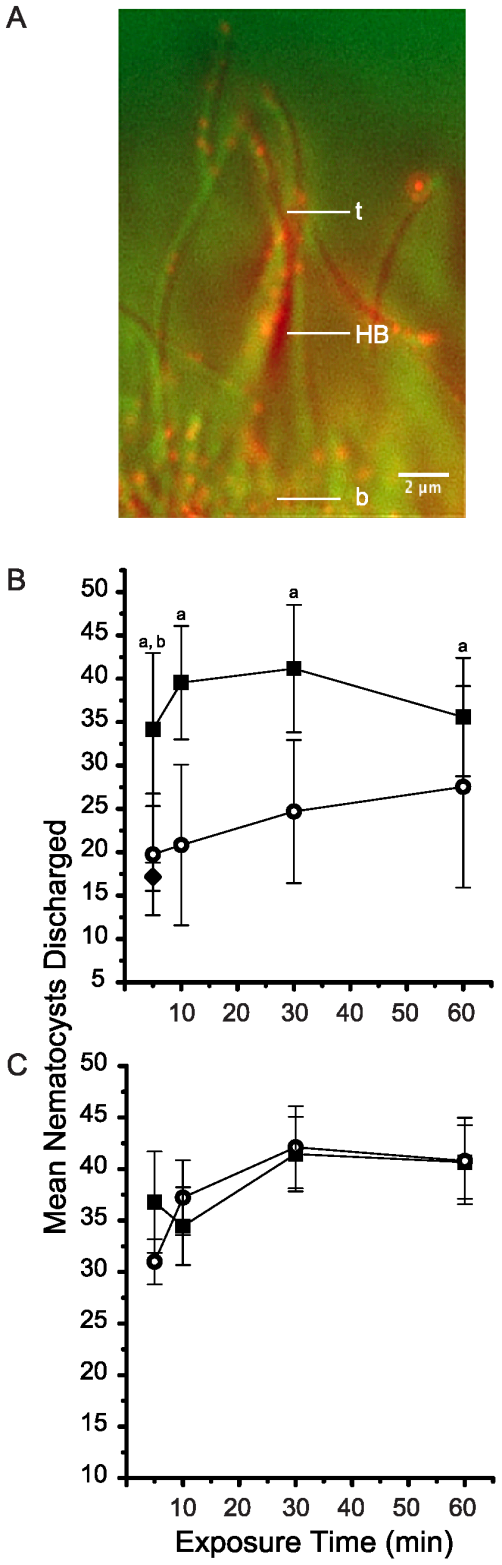
CDH23 immunocytochemistry and effects of CDH23 antibodies on vibration sensitivity in *Nematostella vectensis*. (A) Affinity purified CDH23 antibodies label stereocilia of a hair bundle (HB) with punctate fluorescence. The tip (t) and base (b) of the hair bundle are indicated. The merged image of a hair bundle is shown in transmitted light by oblique contrast (assigned green) and epifluorescence microscopy for CDH23 immunocytochemistry (assigned red). Scale bar  = 2 µm. (B) Vibration sensitivity was evaluated by means of a bioassay based on counting nematocysts discharged into test probes touched to tentacles in the presence of nearby vibrations at a key frequency. Vibration sensitivity was tested at intervals after adding 0.1 nM CDH23 antibodies (final concentration) to the seawater containing intact anemones. The mean number of nematocysts counted per field of view at 400× (±SEM, n = 10–15) is plotted (open circles). Data also are plotted for healthy, vibrating controls (closed squares) and non-vibrating controls (closed diamond) for which the animals were not exposed to exogenously supplied antibodies. (C) Vibration sensitivity was tested at intervals after adding 0.1 nM TRPA1 antibodies (final concentration) as an antibody-loading control. The mean number of nematocysts counted per field of view at 400× (±SEM, n = 5–6) is plotted (open circles). Data also are plotted for healthy, vibrating controls (solid squares). ^a^Significant difference in the mean number of nematocysts discharged between vibrating controls and 0.1 nM CDH23 antibody-treated specimens and ^b^significant difference between the mean number of nematocysts discharged between the vibrating controls and non-vibrating control (*p*<0.05).

The CDH23 antibody labeled hair bundles in *N. vectensis* (Figure 1A) such that punctate fluorescence was observed predominately in the distal half of hair bundle length. The merged image shows an example of a hair bundle imaged in profile (oblique microscopy) decorated with punctate fluorescence (Figure 1A). The punctate fluorescence occurred at a height ranging from 1.1 to 7.8 µm above the base of the hair bundle. The median height of the fluorescence was 3.2 µm above the base of the hair bundle.

### The CDH23 antibody abolishes vibration-dependent discharge of nematocysts and disrupts normal morphology of hair bundles

Vibration sensitivity was tested by touching tentacles of intact anemones with test probes in the presence of nearby vibrations at key frequencies. Healthy controls discharged from 34.1±8.8 to 41.2±7.3 nematocysts into the probes per field of view throughout the experimental period (mean ± standard error of the mean [SEM]; n = 11–14. Henceforth, data are presented as means ± standard error, followed by the sample size where n =  the number of replicate experiments performed). Within 5 min of exposure to 0.1 nM CDH23 antibody, discharge of nematocysts significantly decreased to 19.8±7.0 (n = 12) nematocysts (ANOVA followed by Fisher's post-hoc test, *p* = 0.008*, asterisks indicate a significant difference) and then remained significantly lower than vibrating controls for the remainder of the experiment (Figure 1B). The average number of nematocysts discharged into test probes from the non-vibrating controls (17.2±1.6; n = 7) was approximately a half of that for the vibrating controls. The non-vibrating controls indicated the number of nematocysts discharged in the absence of vibrations. Levels of discharge were significantly different between the non-vibrating controls and vibrating controls (*p* = 7×10^−5^*). On the other hand, there was no significant difference between levels of discharge for non-vibrating controls and CDH23 antibody treated anemones (*p* = 0.393).

At this point, it was important to determine whether the observed effects of the CDH23 antibody on vibration dependent discharge were specific. Accordingly, control experiments were performed in which affinity-purified chicken antibodies raised to a different polypeptide were added to the seawater containing the anemones. The antibody-loading controls consisted of an antibody raised to an intracellular peptide motif of an anemone homolog of TRPA1. While this polypeptide localizes to anemone hair bundles [30], the epitope is predicted to reside in the cytoplasm. The control experiments involved adding 0.1 nM TRPA1 antibody to the seawater and then testing vibration-dependent discharge of nematocysts at specific increments. In the presence of the TRPA1 antibody, levels of discharge did not differ significantly from those for healthy, vibrating controls (*p* = 0.860; Figure1C). The average number of nematocysts discharged by anemones exposed to the TRPA1 antibody was 40.78±4.36 (n = 5–6 at each sampling time). Healthy, vibrating controls discharged a mean of 41.79±5.59 nematocysts (n = 5–6 at each sampling time, Figure 1C).

**Figure 2:**
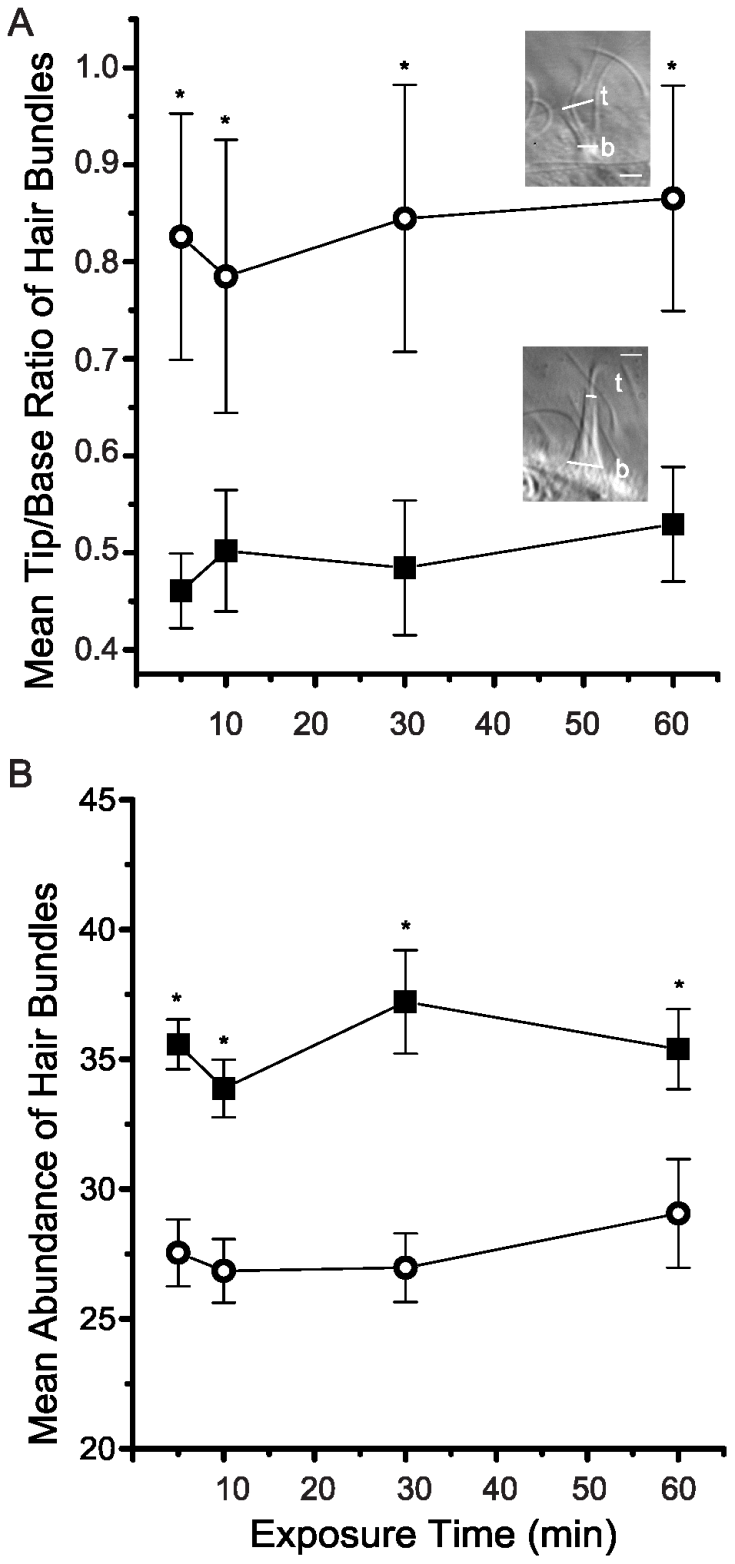
Effects of CDH23 antibodies on morphology and abundance of hair bundles. Morphology was assayed by measuring diameter of hair bundles at their bases (b) and tips (t) and then determining the tip/base ratio for each hair bundle. Hair bundle abundance was measured by the number of hair bundles at the tip of tentacles in a field of view (∼260 µm of tentacle length). (A) The mean tip/base ratio (±SEM, n = 8) is plotted for hair bundles imaged in untreated, healthy controls (closed squares) and in animals exposed to 0.1 nM CDH23 antibodies (open circles). Insets show images of an extremely splayed hair bundle (top) and a normal hair bundle (bottom). Scale bar  = 2 µm. (B) The mean abundance of hair bundles (±SEM, n = 3) on the tentacle epithelium is plotted for untreated, healthy controls (closed squares) and for animals exposed to 0.1 nM CDH23 antibody (open circles). Asterisks indicate significant differences based on time-adjusted pairwise comparisons between controls and experimentals (*p*<0.05).

Because the CDH23 antibody significantly disrupted vibration sensitivity (Figure 1B), possible effects of the CDH23 antibody on morphology of the hair bundles were investigated. In healthy control anemones, hair bundles are conical. Accordingly, a ratio formed by dividing the tip diameter by the base diameter yields a mean of approximately 0.5 (Figure 2A and lower inset). In the presence of 0.1 nM CDH23 antibody, hair bundles significantly splayed, even within 5 min of exposure to the antibody solution, causing the tip/base ratio to nearly double (Figure 2A and upper inset). An example of extremely disarrayed hair bundle that was imaged after exposure to the CDH23 antibody solution is shown in Figure 2A. Time-wise comparisons were all highly significant (*p* = 5×10^−6^*, each based on 8 replicate experiments).

Because the CDH23 antibody caused the hair bundles to significantly splay, we investigated whether such damage might lead to a total disruption of hair bundle structure. Accordingly, we monitored the abundance of hair bundles following exposure to the antibody solution. In healthy controls, the mean abundance of hair bundles was 35.58±0.9 (n = 8) per field of view (∼260 µm of tentacle length). Within 5 min exposure to 0.1 nM CDH23 antibody, the abundance of hair bundles significantly decreased to 27.5±1.3. (*p* = 2×10^−4^*; n = 8). In the continued presence of the CDH23 antibody solution, the mean abundance of hair bundles remained significantly different from that for the healthy control samples (Figure 2B). At this point, it was clear that dilute solutions of the CDH23 antibody disrupt normal structure and function of anemone hair bundles.

We hypothesized that mechanistically, the effects of the CDH23 antibody might either result from: (A) the antibody directly binding to tip links and somehow changing the behavior of the tip links (e.g., by changing their stiffness or overall dimensions), or (B) that the antibody might interfere with protein-protein interactions involving CDH23. We initiated a series of experiments employing the peptide antigen used to generate the CDH23 antibodies described above. Whereas the peptide would not be expected to directly bind tip links, it might directly bind to a protein (or proteins) that normally interact with CDH23 in the hair bundle (as predicted in hypothesis ‘B’).

### The CDH23 peptide binds stereocilia

**Figure 3:**
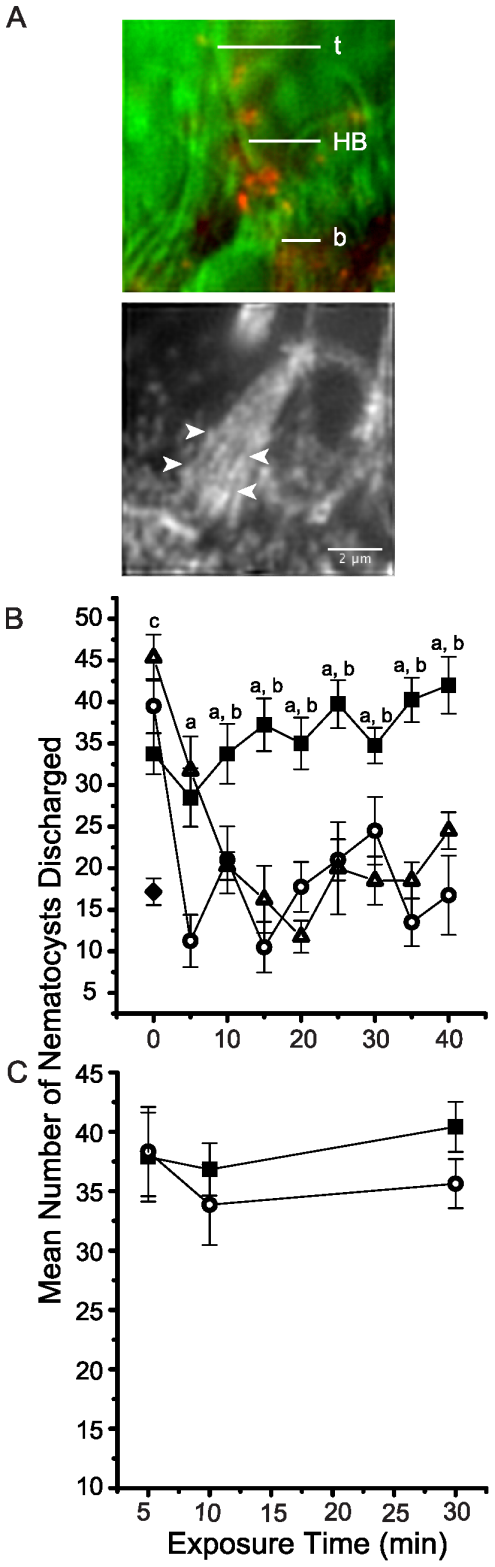
CDH23 peptide cytochemistry and effects of CDH23 peptides on vibration sensitivity in *Nematostella vectensis*. (A) Fluorescently tagged CDH23 peptides label stereocilia near the base of a hair bundle with punctate fluorescence. The upper micrograph depicts a merged image of a hair bundle shown in transmitted light using oblique contrast (assigned green) and epifluorescence microscopy for CDH23 peptide-FITC cytochemistry (assigned red). The tip (t) and base (b) of the hair bundle (HB) are indicated. The lower micrograph depicts immunocytochemistry for TRPN1. It is intended to show the morphology of stereocilia in the hair bundle. White arrowheads indicate the height of the tips of several small diameter stereocilia. Scale bar  = 2 µm. (B) Vibration sensitivity was tested at intervals after adding 0.1 nM CDH23 peptide (open triangles, final concentration) or 10 nM CDH23 peptide (open circles, final concentration) to the seawater containing intact anemones. The mean number of nematocysts counted per field of view (±SEM, n = 6) is plotted for the experimental animals as well as for untreated, vibrating controls (closed squares) and non-vibrating controls (closed diamond). ^a^Significant difference between mean nematocyst discharge for the 10 nM CDH23 peptide treated animals and untreated, vibrating controls. ^b^Significant difference between mean nematocyst discharge for the 0.1 nM CDH23 peptide treated animals and untreated, vibrating controls. ^c^Significant difference between non-vibrating controls and the other three treatments at time 0. (C) Vibration sensitivity was tested at intervals after adding 10 nM harmonin peptide (open circles, final concentration) as a peptide-loading control. The mean number of nematocysts discharged is plotted (±SEM, n = 6–8) for untreated vibrating controls (closed squares) and for animals exposed to the harmonin peptide (open circles). Asterisks indicate significant differences in mean nematocyst discharge (*p*<0.05).

A FITC-conjugated CDH23 peptide was employed to localize its potential binding sites on the tentacle epithelium. The FITC-conjugated CDH23 peptide was observed to bind the hair bundles in *N. vectensis* such that punctate fluorescence was observed predominantly in the proximal half of hair bundle length (upper image in Figure 3A). This observation was verified in five replicate experiments. Punctate fluorescence was observed from 1.2 to 3.2 µm above the base of the hair bundle. The median height of punctate fluorescence was 1.9 µm above the base of the bundle. Although the merged image shows a labeled hair bundle in profile (Figure 3A), it was not possible from these images to evaluate the punctate fluorescence in these micrographs in relation to the position of the tips of stereocilia. We therefore turned our attention to unpublished results of immunocytochemistry for an anemone homolog of TRPN1. These images were selected (lower image in Figure 3A) because they clearly show stereocilia in hair bundles of *N. vectensis*. Small diameter stereocilia range in length from 1.1 to 5.12 µm, with a median length of 1.9 µm. Large diameter stereocilia range in length from 6.5 to 9.1 µm with a median length of 7.9 µm.

### The CDH23 peptide abolishes vibration-dependent discharge of nematocysts

Vibration sensitivity was tested by touching tentacles of intact anemones with test probes in the presence of nearby vibrations at a key frequency. Anemones were incubated in two concentrations of the CDH23 peptide, 0.1 and 10 nM, respectively. Before the CDH23 peptide was added, anemones in the treatment groups discharged nematocysts at levels comparable to those for the healthy, vibrating controls (Figure 3B). The mean number of nematocysts discharged for healthy, vibrating controls was 36.9±3.5 (n = 6) over the experimental period (Figure 3B). The mean number of nematocysts discharged for the non-vibrating control was 17.2±1.6 (n = 7), significantly fewer than for the vibrating controls (*p* = 7×10^−5^*). Within 10 min exposure to 0.1 nM CDH23 peptide, vibration-dependent discharge significantly decreased to a mean of 18.8±1.7 nematocysts (*p* = 0.013*; n = 6) and remained significantly different from healthy controls (*p* = 2×10^−6^−0.002*). Conversely, levels of discharge were comparable for non-vibrating control anemones and anemones exposed to 0.1 nM CDH23 peptide beginning at 10 min exposure (*p* = 0.721) (*p* = 0.100–0.943; Figure 3B).

Increasing the concentration of the CDH23 peptide by 100 fold increased its potency. Whereas a 5 min exposure to 0.1 nM CDH23 peptide failed to decrease the vibration-dependent discharge, a 5 min of exposure to 10 nM CDH23 peptide decreased the mean number of nematocysts discharged significantly to 12.5±3.3 (*p* = 1×10^−6^*; n = 6). The mean number of nematocysts discharged thereafter remained significantly different from that for the vibrating controls (Figure 3B). Furthermore, the mean number of nematocysts discharged from anemones exposed to 10 nM CDH23 peptide was comparable to that for the non-vibrating controls (*p* = 0.178–0.917) for the entire experiment.

In order to test the specificity of the effects of the CDH23 peptide on vibration dependent discharge of nematocysts, we added a different peptide to the seawater containing the anemones. A 17 amino acid peptide of a *N. vectensis* homolog of harmonin, corresponding to residues 320–336, was used as a peptide-loading control (Figure 3C). Harmonin is a cytoplasmic protein that is important to normal function in hair cells [32], [33]. In the presence of vibrations at a key frequency, the mean number of nematocysts discharged was 38.4±2.7 (n = 6–8) in healthy, vibrating controls, and 35.9±3.1 (n = 6–8) for anemones tested in the presence of 10 nM harmonin peptide. Anemones exposed to 10 nM harmonin peptide showed levels of nematocyst discharge comparable to those for healthy, vibrating controls (*p* = 0.628) for the entire experimental period (Figure 3C).

### The CDH23 peptide decreases the mechanotransduction current

**Figure 4:**
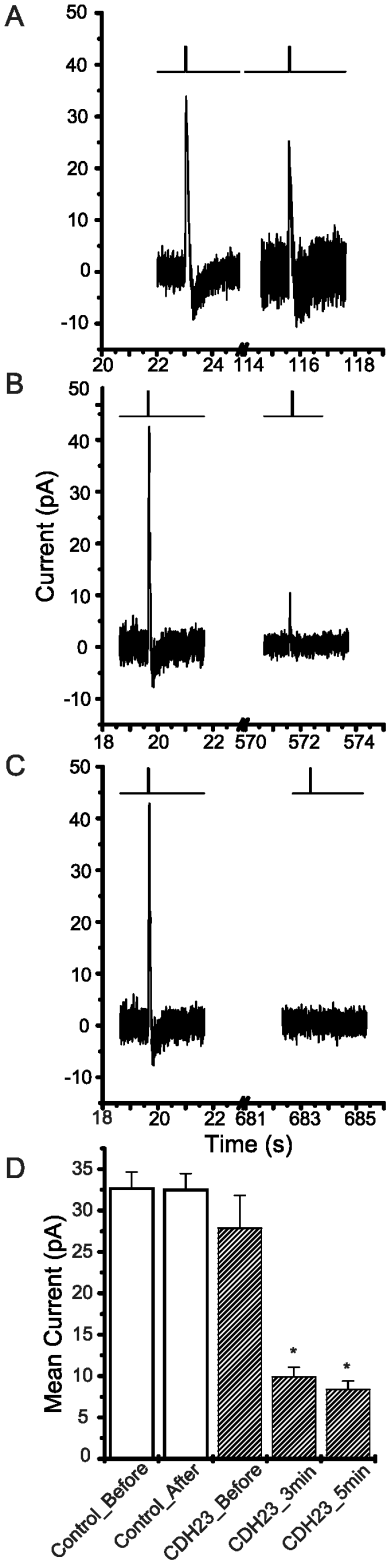
Effects of CDH23 peptides on the mechanoelectric responses induced by deflecting hair bundles. Hair bundles were deflected by pressure jets delivered by a nearby puffer pipette. Recording pipettes were attached to the plasma membrane adjacent to the base of the hair bundles. The TTL signal depicting the opening and closing the solenoid valve controlling pressure delivered to a puffer pipet is shown above representative traces depicting membrane current transients induced by hair bundle deflection. (A) Recordings are shown for a perfusion control before (left) and after perfusion of potassium-enriched seawater (right). (B) Recordings are shown before (left) and 3 min after the perfusion of potassium-enriched seawater containing 10 nM CDH23 peptide (right). (C) Recordings are shown before (left) and 5 min after the perfusion of potassium-enriched seawater containing 10 nM CDH23 peptide (right). (D) The mean peak membrane current induced by deflecting hair bundles was calculated and plotted for before and after perfusion (±SEM, n = 10). Perfusion controls (empty bar) had perfusion of potassium-enriched seawater only. The CDH23 peptide-treated specimens (hatched bar) were exposed to potassium-enriched seawater containing 10 nM CDH23 peptide (final concentration). The peak membrane current induced by deflecting hair bundles was measured at two sampling times after the perfusion, 3 min and 5 min, in the CDH23 peptide-treated specimens. Asterisks indicate significant differences in mean peak membrane current between before and after perfusion (*p*<0.05).

We next tested whether the CDH23 peptide might directly affect mechanotransduction of hair bundles by using electrophysiology. First, positive control experiments were conducted in order to rule out significant effects of perfusion on mechanotransduction. A representative recording indicates strong current transients were induced by deflecting a hair bundle before and after perfusion of potassium enriched seawater (Figure 4A). The mean peak currents induced by bundle deflection were 32.68±1.95 pA (n = 10) before perfusion and 32.52±1.94 pA (n = 10) after perfusion of potassium-enriched seawater lacking the peptide (Figure 4D). For this control experiment, there was no significant difference between the mean peak currents recorded before perfusion as compared to those recorded after perfusion (*p* = 0.940).

**Figure 5:**
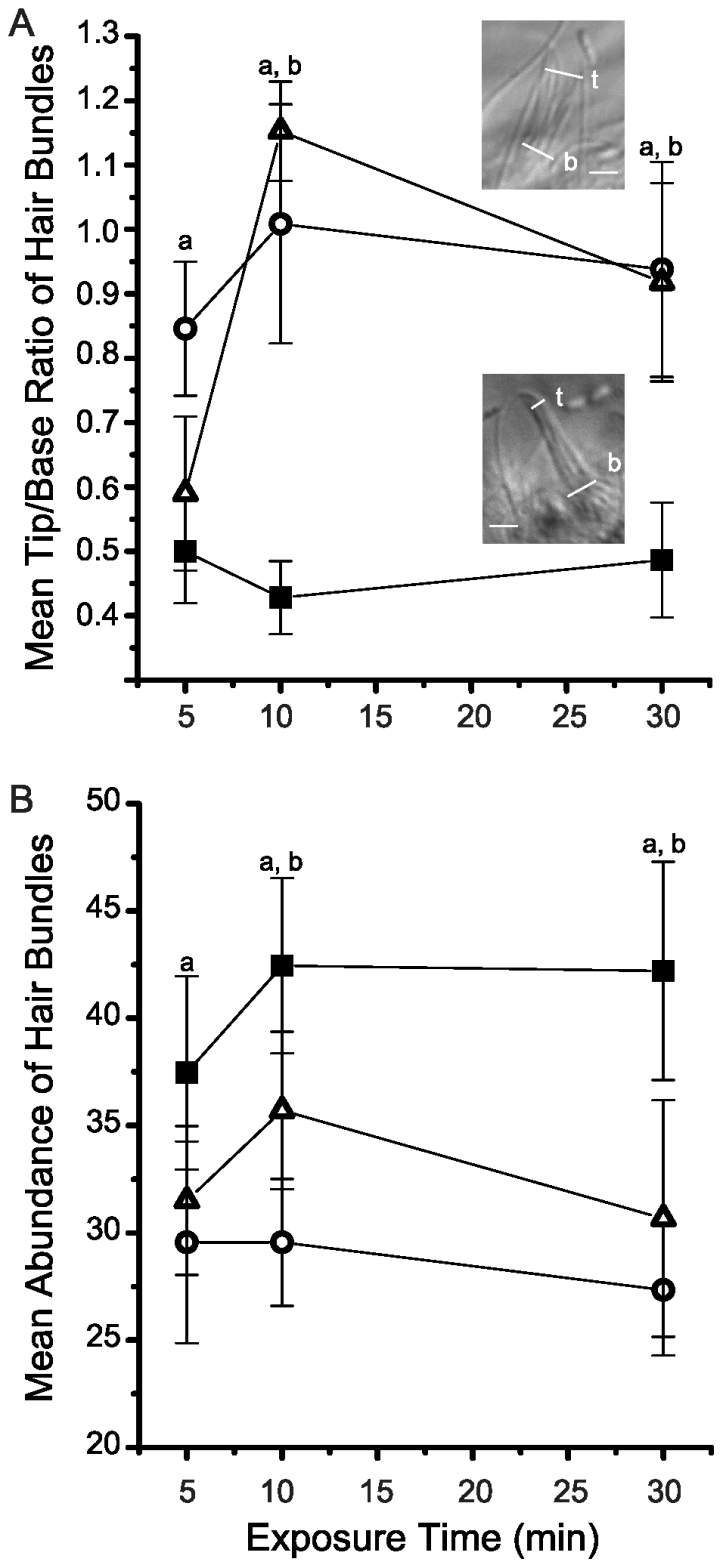
Effects of the CDH23 peptide on morphology and abundance of hair bundles. Hair bundles were evaluated in untreated, healthy controls (closed squares), in animals exposed to 0.1 nM CDH23 peptide (open triangles) or to 10 nM CDH23peptide (open circles). (A) Morphology was assayed by the tip/base ratio. The mean tip/base ratio (±SEM, n = 5–8) is plotted. Insets show representative images of an extremely splayed hair bundle (top) and normal hair bundle (bottom). The diameters of tip (t) and base (b) are indicated. Scale bar  = 2 µm. (B) The mean abundance of hair bundles; ±SEM, n = 3) along the tentacle epithelium is plotted. ^a^Significant difference between data for the 10 nM CDH23 peptide treated specimens and untreated controls and ^b^Significant difference between data for the 0.1 nM CDH23 peptide treated specimens and untreated controls.

A 10 nM CDH23 peptide solution was perfused into the chamber containing the specimen. A representative recording indicates strong current transients were induced by bundle deflection before perfusion but not 3 min after perfusion (Figure 4B) or 5 min after perfusion (Figure 4C). Before perfusing the CDH23 peptide solution, mean peak currents induced by bundle deflection were 27.89±3.92 pA (n = 10). Interestingly, 3 min after perfusing the10 nM CDH23 peptide solution, the mean peak currents significantly decreased to 9.92±1.13 pA (n = 10; *p* = 2×10^−5^*; Figure 4D). At 5 min after perfusion, the mean peak currents further decreased to 8.40±1.00 pA (n = 10), also significantly different from mean peak currents recorded before perfusion (*p* = 5×10^−6^*; Figure 4D).

### The CDH23 peptide disrupts normal morphology of the hair bundles

Because the CDH23 peptide disrupts hair bundle function, possible effects of exposure to the CDH23 peptide on morphology of hair bundles were investigated. Hair bundles of healthy controls are conical in shape (Figure 5A and lower inset). The mean tip/base ratio of hair bundles in controls was 0.47±0.08 (n = 5–8) throughout the experimental period. After 5 min exposure to 10 nM CDH23 peptide, hair bundles splayed and the mean tip/base ratios significantly increased to 0.85±1.03 (*p* = 0.046*; n = 8). At the lower CDH23 peptide concentration of 0.1 nM, hair bundles splayed and the mean tip/base ratios of hair bundles significantly increased, but not until 10 min of exposure (1.15±0.08; *p* = 2×10^−6^*; n = 8) (Figure 5A). In order to examine the possibility that the CDH23 peptide completely disrupts hair bundle integrity, we investigated whether the CDH23 peptide changes the abundance of hair bundles on the tentacle epithelium (Figure 5B).

The mean abundance of hair bundles was between 37.5±4.5 and 42.4±4.1 for healthy controls throughout the experimental period. At five minutes exposure to 10 nM CDH23 peptide, the mean abundance of hair bundles significantly decreased to (29.6±3.1; n = 3; *p* = 0.018*) approximately ¾ of that for controls (42.4±4.2; n = 3). On the other hand, the mean abundance of hair bundles at five minutes of exposure to 0.1 nM CDH23 peptide (31.5±3.4; n = 3) was slightly higher and, consequently, insignificantly different from the untreated controls (*p* = 0.065). Nevertheless, both concentrations of the CDH23 peptide significantly decreased mean abundance of hair bundles after 30 min of exposure (*p* = 3×10^−5^−0.047*; Figure 5B).

### The CDH23 peptide decreases F-actin in stereocilia

**Figure 6:**
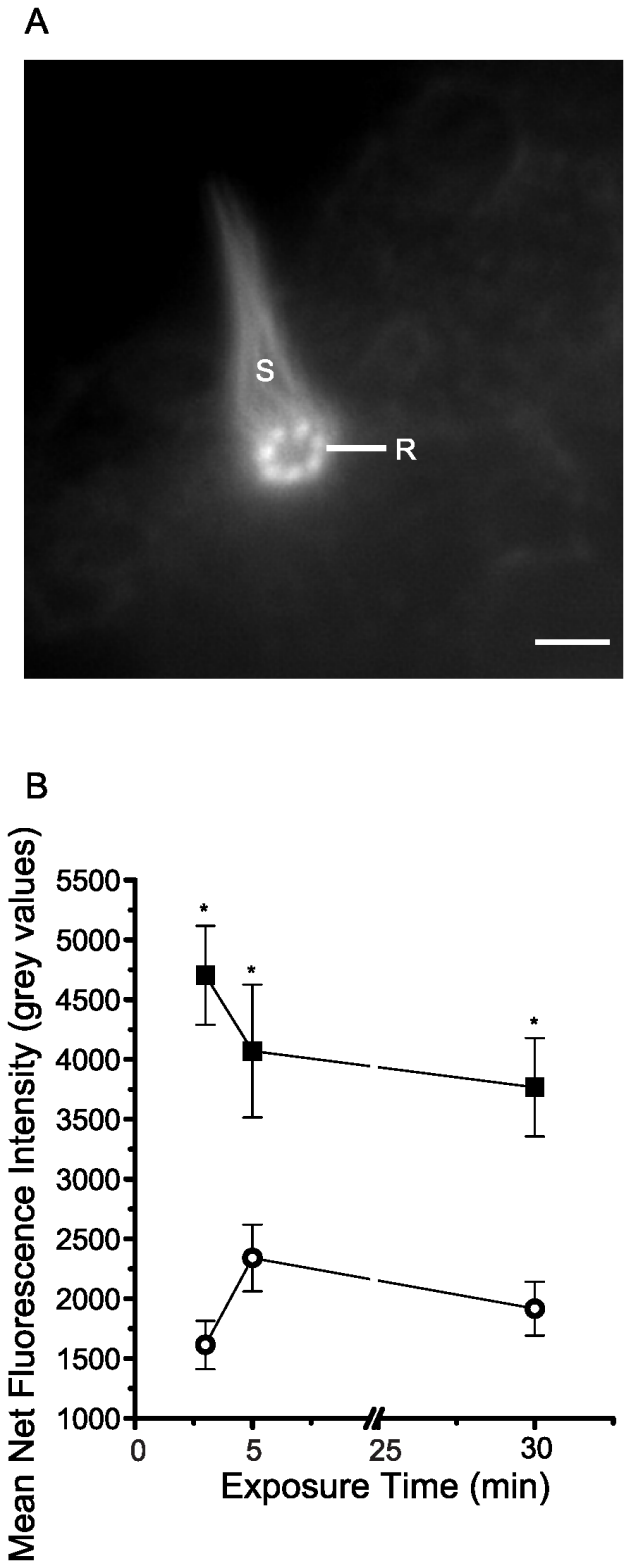
F-actin cytochemistry and effects of the CDH23 peptide on rhodamine-phalloidin fluorescence intensity of hair bundles. (A) Rhodamine-phalloidin labeled stereocilia (S) and rootlets (R) of hair bundles in *N. vectensis*. Scale bars  = 2 µm. (B) Untreated controls (closed squares) and specimens incubated in 10 nM CDH23 peptide (open circles) were fixed and then incubated in a rhodamine conjugate of phalloidin. Mean fluorescence intensity was obtained from analysis of digital images of hair bundles. Background fluorescence was subtracted to yield net fluorescence intensity. Mean net fluorescence intensity is plotted (±SEM, n = 3) in grey values. Asterisks indicate significant differences between mean net fluorescence intensity of untreated controls and specimens exposed to 10 nM CDH23 peptide (*p*<0.05).

It was intriguing to observe relatively dramatic effects of the CDH23 peptide on hair bundle morphology. Therefore, we investigated whether the CDH23 peptide might affect actin in stereocilia. Stereocilia and rootlets of stereocilia were labeled with rhodamine phalloidin (Figure 6A). The fluorescence intensity of rhodamine phalloidin in stereocilia was compared between untreated, healthy controls and specimens exposed to the CDH23 peptide. Background fluorescence was subtracted from images of phalloidin labeled stereocilia. The mean net fluorescence intensity of rhodamine phalloidin in untreated controls was 4180.5±460.1 grey values (n = 3 at each sampling time) throughout the entire experimental period (Figure 6B). Specimens fixed at three min of exposure to 10 nM CDH23 peptide exhibited stereocilia that were significantly less brightly fluorescent (*p* = 1×10^−6^*), having a mean net intensity of 1614.3±202.5 grey values (n = 3). The fluorescence intensity of rhodamine phalloidin in peptide-treated stereocilia was consistently less than half of that in untreated controls for the rest of experimental period (*p* = 1×10^−4^*; Figure 6B).

## Discussion

CDH23 is an important component of tip links, extracellular linkages that participate in mechanotransduction of hair cells in vertebrate animals [16], [18], [34]. Disruption of the structural integrity of CDH23 by exposing hair cells to calcium depleted buffers [20], or by the enzymatic digestion of tip links by elastase [35] leads to a loss of mechanotransduction. In addition, known mutations in the sequence of genes encoding CDH23 lead to deafness syndromes [36], [37].

In the present study, using an invertebrate model system, we found that CDH23 likely participates in dynamic protein-protein interactions critical to normal hair cell structure and function. According to the model, CDH23 in tip links cyclically binds and dissociates from specific polypeptides over a time course of a few minutes. At the center of this hypothesis is an exoplasmic C-terminal motif of CDH23 that evidently is unique to the anemone system. At 0.1 nM concentrations, affinity purified CDH23 antibodies raised to this motif disrupted vibration sensitivity when added to seawater in *N. vectensis* (this study) and another anemone species, *Haliplanella luciae* [28]. Thus, the disruption of vibration sensitivity caused by the CDH23 antibody is achieved at very low antibody concentrations. This disruptive effect appears to be specific because a different affinity purified antibody raised to an intracellular peptide antigen of TRPA1 did not disrupt vibration sensitivity. Furthermore, the CDH23 antibodies label hair bundles in *N. vectensis* as well as in *Haliplanella luciae* [28]. Evidently, the antibody binds CDH23 at this exoplasmic motif where it interferes with dynamic protein-protein interactions that involve the motif. Nevertheless, because antibodies can generate spurious results [e.g., 38], we also tested the model in experiments in which the antigenic CDH23 peptide was used rather than the CDH23 antibody. Whereas the CDH23 antibody is presumed to mask the C-terminus of cadherin 23, the CDH23 peptide imitates the exoplasmic motif of cadherin 23 and is presumed to bind to one or more polypeptides in hair bundles, the identity of which is (are) unknown. Henceforth, we will refer to such polypeptides as ‘cadherin binding proteins’ or ‘CBPs’. Consistent with the model is the finding that a disruption of vibration sensitivity also occurred in the presence of low concentrations, 0.1 to 10 nM, of the CDH23 peptide. Likewise, the disruptive effect induced by the CDH23 peptide appears to be specific because a different peptide corresponding to a portion of harmonin failed to disrupt vibration sensitivity. Finally, we confirmed that the CDH23 peptide disrupts mechanotransduction using electrophysiology. According to the model, as CDH23 cyclically dissociates from CBPs, free exoplasmic domains of CDH23 are available to bind exogenously supplied CDH23 antibodies. Likewise, as CDH23 cyclically dissociates from CBPs, CDH23 binding domains are available in CBPs to bind exogenously supplied CDH23 peptides. Upon binding exogenously supplied CDH23 antibodies (or CDH23 peptides), normal protein-protein interactions involving CDH23 and CBPs are disrupted leading to a loss of mechanotransduction. Thus, mechanotransduction requires normal protein-protein interactions between CDH23 and CBPs. Presumably, CDH23 binds CBPs with high affinity. Dissociation may be driven primarily by mechanical tension tending to pull the CDH23/CBP protein complexes apart. Antibodies raised to the CDH23 C-terminal motif label hair bundles at positions ranging from 1.1 to 7.8 µm above the base of the hair bundle. Given that small diameter stereocilia originating from the hair cells range in length from 1.1 to 5.2 µm, it is likely that CDH23 occurs both on large diameter stereocilia originating from the sensory neuron and small diameter stereocilia originating from the surrounding hair cells.

Several experimental constraints made localizing CBPs difficult. These experiments incorporated a FITC-conjugated CDH23 peptide. First, labeling was sensitive to fixation such that labeling was absent in tissue that was fixed prior to incubating the tissue in the FITC-conjugated peptide. Second, because the peptide disrupts normal morphology of hair bundles, incubation times were kept brief (i.e., too brief to permit a significant disruption of normal morphology). Third, because binding between CDH23 and CBPs may be critical to maintaining structure and function of the hair bundles, the occurrence of ‘free’ CBPs (i.e., those that normally bind CDH23 but occasionally dissociate from it) may be relatively rare. Such rare ‘free’ CBPs would be a requirement for the fluorescent peptide to label hair bundles. As a result of these constraints, labeled hair bundles were in a small minority. In a few cases, we were able to observe the FITC-conjugated CDH23 peptide to label hair bundles imaged in profile. In such cases, the label occurred from 1.2 to 3.2 µm above the base of the hair bundle. Indeed, the median peptide label at 1.9 µm above the base of the hair bundle agrees with our estimates of the median length of stereocilia originating from the hair cells, also at 1.9 µm. Thus, based on limited observations, it appears that the CBPs, polypeptides in hair bundles that bind CDH23, may occur at or near the tips of small diameter stereocilia originating from the hair cells. If this hypothesis is correct, then it may also explain why the CDH23 immunocytochemistry preferentially labels more distally in the hair bundles. If the CBPs are restricted to the hair cells, as was proposed above, then the CDH23s in neuronal stereocilia may well have free exoplasmic C-termini available to bind the CDH23 antibodies. Although CDH23 immunocytochemistry was observed from 1.1 to 7.8 µm above the base of the hair bundle, and thus overlaps with the distribution of CBPs, the median label was observed at 3.2 µm above the base of the hair bundle. As an alternative explanation, the relatively poor performance of the FITC-conjugated peptide to label hair bundles, despite the robust activity of the unlabeled peptide to disrupt normal morphology and mechanotransduction of the hair bundles, may result from the bulky FITC moiety (MW = 398 Da) at approximately three times the mass of an amino acid partially interfering with peptide-binding to CBPs.

In comparison to the results presented in this study, other treatments known to disrupt function of hair bundles in sea anemones require relatively high doses in order to do so. For example, 0.1 mM concentrations of streptomycin or ruthenium red are required to abolish vibration sensitivity, presumably by interacting with the mechanotransduction channel [23], [27], [30]. Thus, the CDH23 antibodies or CDH23 peptides are approximately 10^6^ times more effective than are streptomycin and ruthenium red in disrupting mechanosensitivity of the hair bundles. From the standpoint of kinetics, we find that at the lowest dose tested, 0.1 nM, the CDH23 peptide required more than 5 min to disrupt hair bundle structure and function. At 10 nM, the CDH23 peptide disrupted mechanosensitivity within approximately 3 min of exposure (the earliest time point tested).

It is intriguing to consider the observation that exogenously supplied CDH23 peptide disrupts normal morphology of the hair bundles within a matter of a few minutes while significantly decreasing levels of F-actin in stereocilia. Previous studies showed actin to be dynamic in hair bundles in sea anemones. First, sea anemone hair bundles normally elongate, shorten, or otherwise reorganize stereocilia in the hair bundle [39] in response to specific prey odorants as part of a system in which the hair bundles are dynamically tuned to respond to the swimming movements of prey. Whereas elongation requires polymerization of actin in stereocilia, shortening requires depolymerization of actin [26]. Second, a reorganization of actin in stereocilia was observed during self-repair of anemone hair bundles occurring after acute trauma. Specifically, after the trauma, F-actin was first partially depolymerized and then was repolymerized before the hair bundle fully recovered. The time course of actin repolymerization in stereocilia coincided with the time course for the recovery of the mechanoelectric response [40].

Perhaps the loss of F-actin from stereocilia is initiated by the hair cell in response to the loss of mechanotransduction. Following trauma, anemone hair bundles self-repair in a mechanism that involves secreted proteins [41] and that includes a decrease in F-actin in stereocilia that recovers to normal at the completion of repair [40]. Alternatively, it also is possible that by disrupting normal protein-protein interactions between CDH23 and CBPs, regulation of F-actin in stereocilia is disrupted leading to a disruption of normal morphology. As a consequence, mechanotransduction is lost.

This study showed that dynamic protein-protein interactions likely occur between a unique exoplasmic C-terminal motif of CDH23 and putative cadherin binding proteins (CBPs) on stereocilia. Furthermore, such interactions are crucial for normal mechanotransduction in hair cells of an invertebrate model system, *N. vectensis*. The elucidation of CBPs in anemone hair bundles in stereocilia and the biological consequences of such interactions will provide new insight as to how hair bundles function in anemones as well as illuminating the functions of CDH23 in perhaps the ‘earliest’ hair bundles to appear in the course of evolution.

## References

[1] Tilney LG, Cotanche TA, Tilney MS (1992) Actin filaments, stereocilia and hair cells of the bird cochlea. Development 116: 213–226 PMID: 1483389. 1483389 10.1242/dev.116.1.213

[2] Hudspeth AJ (1985) The cellular base of hearing: the biophysics of hair cells. Science 23: 745–752 10.1126/science.2414845 2414845

[3] Hudspeth AJ (1989) How the ear's works work. Nature 341: 397–404 10.1038/341397a0 2677742

[4] Gillespie PG, Müller U (2009) Mechanotransduction by hair cells: models, molecules, and mechanisms. Cell 139: 33–44 10.1016/j.cell.2009.09.010 19804752 PMC2888516

[5] Hudspeth AJ (1982) Extracellular current flow and the site of transduction by vertebrate hair cells. J Neurosci 2: 1–10 PMID: 6275046. 6275046 10.1523/JNEUROSCI.02-01-00001.1982 PMC6564293

[6] Jaramillo F, Hudspeth AJ (1991) Localization of the hair cell's transduction channels at the hair bundle's top by iontophoretic application of a channel blocker. Neuron 7: 409–420 10.1016/0896-6273(91)90293-9 1716929

[7] Denk W, Holt JR, Shepherd GM, Corey DP (1995) Calcium imaging of single stereocilia in hair cells: localization of transduction channels at both ends of tip links. Neuron 15: 1311–1321 10.1016/0896-6273(95)90010-1 8845155

[8] Beurg M, Fettiplace R, Nam J-H, Ricci AJ, Inserm U, et al (2009) Localization of inner hair cell mechanotransducer channers using high speed calcium imaging. Nat Neurosci 12: 553–558 10.1038/nn.2295 19330002 PMC2712647

[9] Eatock RA, Corey DP, Hudspeth AJ (1987) Adaptation of mechanoelectrical transduction in hair cells of the bullfrog's sacculus. J Neurosci 7: 2821–2836 PMID: 3498016 3498016 10.1523/JNEUROSCI.07-09-02821.1987 PMC6569155

[10] Howard J, Hudspeth AJ (1987) Mechanical relaxation of the hair bundle mediates adaptation in mechanoelectrical transduction by the bullfrog's saccular hair cell. Proc Natl Acad Sci USA 84: 3064–3068 PMID: 3495007. 3495007 10.1073/pnas.84.9.3064 PMC304803

[11] Hudspeth AJ, Corey DP (1977) Sensitivity, polarity, and conductance change in the response of vertebrate hair cells to controlled mechanical stimuli. Proc Natl Acad Sci USA 74: 2407–2411 PMID: 329282. 329282 10.1073/pnas.74.6.2407 PMC432181

[12] Pickles JO, Comis SD, Osborne MP (1984) Cross-links between stereocilia in the guinea pig organ of Corti, and their possible relation to sensory transduction. Hear Res 15: 103–112 10.1016/0378-5955(84)90041-8 6436216

[13] Howard J, Roberts W, Hudspeth AJ (1988) Mechanoelectrical Transduction by hair cells. Annu Rev Biophys Biomol Struct 17: 99–124 10.1146/annurev.bb.17.060188.000531 3293600

[14] Kachar B, Parakkal M, Kurc M, Zhao Y, Gillespie PG (2000) High-resolution structure of hair-cell tip links. Proc Natl Acad Sci USA 97: 13336–13341 10.1073/pnas.97.24.13336 11087873 PMC27225

[15] Tsuprun V, Goodyear RJ, Richardson GP (2004) The structure of tip links and kinocilial links in avian sensory hair bundles. Biophys J 87: 4106–4112 10.1529/biophysj.104.049031 15377520 PMC1304919

[16] Siemens J, Lillo C, Dumont RA, Reynolds A, Williams DS, et al (2004) Cadherin 23 is a component of the tip link in hair-cell stereocilia. Nature 428: 950–955 10.1038/nature02483 15057245

[17] Kazmierczak P, Sakaguchi H, Tokita J, Wilson-Kubalek EM, Milligan RA, et al (2007) Cadherin 23 and protocadherin 15 interact to form tip-link filaments in sensory hair cells. Nature 449: 87–92 10.1038/nature06091 17805295

[18] Söllner C, Rauch G, Siemens J, Geisler R, Schuster SC, et al (2004) Mutations in cadherin 23 affect tip links in zebrafish sensory hair cells. Nature 428: 955–959 10.1038/nature02484 15057246

[19] Di Palma F, Pellegrino R, Noben-Trauth K (2001) Genomic structure, alternative splice forms and normal and mutant alleles of cadherin 23 (Cdh23). Gene 281: 31–41 10.1016/S0378-1119(01)00761-2 11750125

[20] Assad JA, Shepherd GM, Corey DP (1991) Tip-link integrity and mechanical transduction in vertebrate hair cells. Neuron 7: 985–994 10.1016/0896-6273(91)90343-X 1764247

[21] Gale JE, Marcotti W, Kennedy HJ, Kros CJ, Richardson GP (2001) FM 1-43 dye behaves as a permanent blocker of the hair-cell mechanotransducer channel. J Neurosci 21: 7013–7025 PMID: 11549711. 11549711 10.1523/JNEUROSCI.21-18-07013.2001 PMC6762973

[22] Sotomayor M, Corey DP, Schulten K (2005) In search of the hair-cell gating spring: elastic properties of ankyrin and cadherin repeats. Structure 13: 669–682 10.1016/j.str.2005.03.001 15837205

[23] Watson GM, Mire P, Hudson RR (1997) Hair bundles of sea anemones as a model system for vertebrate hair bundles. Hear Res 107: 53–66 10.1016/S0378-5955(97)00022-1 9165347

[24] Watson GM, Hessinger DA (1989) Cnidocyte mechanoreceptors are tuned to the movements of swimming prey by chemoreceptors. Science 243: 1589–1591 10.1126/science.2564698 2564698

[25] Watson GM, Mire P, Hudson RR (1998) Frequency specificity of vibration dependent discharge of nematocysts in sea anemones. J Exp Zool 281: 582–593 doi:10.1002/(SICI)1097-010X(19980815)281:6<582::AID-JEZ6>3.0.CO;2-J 9697323

[26] Watson GM, Roberts J (1995) Chemoreceptor-mediated polymerization and depolymerization of actin in hair bundles of sea anemones. Cell Motil Cytoskeleton 30: 208–220 PMID: 7758137. 7758137 10.1002/cm.970300305

[27] Mire P, Watson GM (1997) Mechanotransduction of hair bundles arising from multicellular complexes in anemones. Hear Res 113: 224–234 10.1016/S0378-5955(97)00145-7 9388001

[28] Watson GM, Pham L, Graugnard EM, Mire P (2008) Cadherin 23-like polypeptide in hair bundle mechanoreceptors of sea anemones. J Comp Physiol [A] 194: 811–820 10.1007/s00359-008-0352-0 18654787

[29] Watson GM, Mire P, Kinler KM (2009) Mechanosensitivity in the model sea anemone *Nematostella vectensis* . Mar Biol 156: 2129–2137 10.1007/s00227-009-1243-9

[30] Mahoney JL, Graugnard EM, Mire P, Watson GM (2011) Evidence for involvement of TRPA1 in the detection of vibrations by hair bundle mechanoreceptors in sea anemones. J Comp Physiol [A] 197: 729–742 10.1007/s00359-011-0636-7 21394510

[31] Mire-Thibodeaux P, Watson GM (1994) Morphodynamic hair bundles arising from sensory cell/supporting cell complexes frequency-tune nematocyst discharge in sea anemones. J Exp Zool 268: 282–292 10.1002/jez.1402680404 8195744

[32] Verpy E, Leibovici M, Zwaenepoel I, Liu XZ, Gal A, et al (2000) A defect in harmonin, a PDZ-domain containing protein expressed in the inner ear sensory hair cells, underlies Usher syndrome type 1C. Nature 26: 51–55 10.1038/79171 10973247

[33] Boëda Batiste, El-amraoui Aziz, Bahloul Amel, Goodyear R, Blanchard Â, et al (2002) Myosin VIIa, harmonin and cadherin 23, three Usher I gene products that cooperate to shape the sensory hair cell bundle. EMBO J 21: 6689–6699 10.1093/emboj/cdf689 12485990 PMC139109

[34] Müller U (2008) Cadherins and mechanotransduction by hair cells. Curr Opin Cell Biol 20: 557–566 10.1016/j.ceb.2008.06.004 18619539 PMC2692626

[35] Meyer J, Furness DN, Zenner HP, Hackney CM, Gummer AW (1998) Evidence for opening of hair-cell transducer channels after tip-link loss. J Neurosci 18: 6748–6756 PMID: 9712646 9712646 10.1523/JNEUROSCI.18-17-06748.1998 PMC6792952

[36] Bolz H, von Brederlow B, Ramírez A, Bryda EC, Kutsche K, et al (2001) Mutation of CDH23, encoding a new member of the cadherin gene family, causes Usher syndrome type 1D. Nat Genet 27: 108–112 10.1038/83667 11138009

[37] Bork JM, Peters LM, Riazuddin S, Bernstein SL, Ahmed ZM, et al (2001) Usher syndrome 1D and nonsyndromic autosomal recessive deafness DFNB12 are caused by allelic mutations of the novel cadherin-like gene CDH23. Am J Hum Genet 68: 26–37 10.1086/316954 11090341 PMC1234923

[38] Saper CB (2005) An open letter to our readers on the use of antibodies. J Comp Neurol 493: 477–478 10.1002/cne.20839 16304632

[39] Mire P, Nasse J (2002) Hair bundle motility induced by chemoreceptors in anemones. Hear Res 163: 111–120 10.1016/S0378-5955(01)00392-6 11788205

[40] Watson GM, Mire P (2002) Reorganization of actin during repair of hair bundle mechanoreceptors. J Neurocytol 30: 895–906 PMID: 12373097. 10.1023/a:1020665116719 12373097

[41] Watson GM, Mire P, Hudson R (1998) Repair of hair bundles in sea anemones by secreted proteins. Hear Res 115: 119–128 10.1016/S0378-5955(97)00185-8 9472741

